# 5-(Prop-2-yn­yl)-5*H*-dibenzo[*b*,*f*]azepine

**DOI:** 10.1107/S1600536812007866

**Published:** 2012-03-17

**Authors:** S. Yousuf, M. Khan, S. Fazal, M. Butt, F. Z. Basha

**Affiliations:** a HEJ Research Institute of Chemistry, International Center for Chemical and Biological Sciences, University of Karachi, Karachi 75270, Pakistan

## Abstract

The asymmetric unit of the title compound, C_17_H_13_N, contains two independent butterfly-shaped mol­ecules. The seven-membered azepine rings both adopt a boat conformation. The dihedral angles between the benzene rings in the two mol­ecules are 46.95 (11) and 52.21 (11)°.

## Related literature

For the biological activities of imino­stilbene, see: Kumar & Naik (2010 ▶); Balaure *et al.* (2009 ▶); Bhatt & Patel (2005 ▶); Fuenfschilling *et al.* (2005 ▶); Rosowsky *et al.* (2004 ▶); Brzozowski & Saczewski (2002 ▶); Kulkarni *et al.* (1991 ▶); Arya *et al.* (1977 ▶). For the crystal structures of the closely related compounds, see: Jayasankar *et al.* (2009 ▶); Nagaraj *et al.* (2005 ▶); Sadashiva *et al.* (2005 ▶).
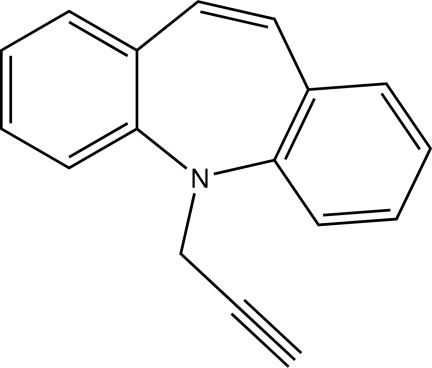


## Experimental

### 

#### Crystal data

C_17_H_13_N
*M* *_r_* = 231.28
Monoclinic, 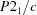

*a* = 11.4406 (5) Å
*b* = 10.0256 (4) Å
*c* = 22.3155 (10) Å
β = 92.910 (1)°
*V* = 2556.26 (19) Å^3^
*Z* = 8
Mo *K*α radiation
μ = 0.07 mm^−1^
*T* = 273 K
0.36 × 0.19 × 0.15 mm

#### Data collection

Bruker SMART APEX CCD area-detector diffractometer
Absorption correction: multi-scan (*SADABS*; Bruker, 2000 ▶) *T*_min_ = 0.975, *T*_max_ = 0.990
14791 measured reflections
4760 independent reflections
2894 reflections with *I* > 2σ(*I*)
*R*_int_ = 0.031

#### Refinement

*R*[*F* ^2^ > 2σ(*F* ^2^)] = 0.048
*wR*(*F* ^2^) = 0.129
*S* = 1.02
4760 reflections
333 parameters
H atoms treated by a mixture of independent and constrained refinement
Δρ_max_ = 0.13 e Å^−3^
Δρ_min_ = −0.17 e Å^−3^

### 

Data collection: *SMART* (Bruker, 2000 ▶); cell refinement: *SAINT* (Bruker, 2000 ▶); data reduction: *SAINT*; program(s) used to solve structure: *SHELXS97* (Sheldrick, 2008 ▶); program(s) used to refine structure: *SHELXL97* (Sheldrick, 2008 ▶); molecular graphics: *SHELXTL* (Sheldrick, 2008 ▶); software used to prepare material for publication: *SHELXTL*, *PARST* (Nardelli, 1995 ▶) and *PLATON* (Spek, 2009 ▶).

## Supplementary Material

Crystal structure: contains datablock(s) global, I. DOI: 10.1107/S1600536812007866/rz2705sup1.cif

Structure factors: contains datablock(s) I. DOI: 10.1107/S1600536812007866/rz2705Isup2.hkl

Supplementary material file. DOI: 10.1107/S1600536812007866/rz2705Isup3.cml

Additional supplementary materials:  crystallographic information; 3D view; checkCIF report

## Figures and Tables

**Fig. 1.**
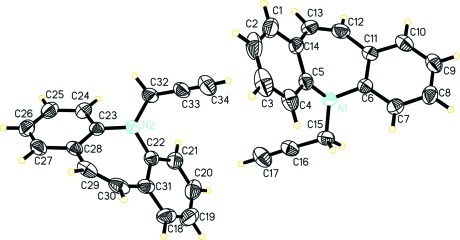
The molecular structure of the title compound with displacement ellipsoids drawn at the 30% probability level.

**Fig. 2.**
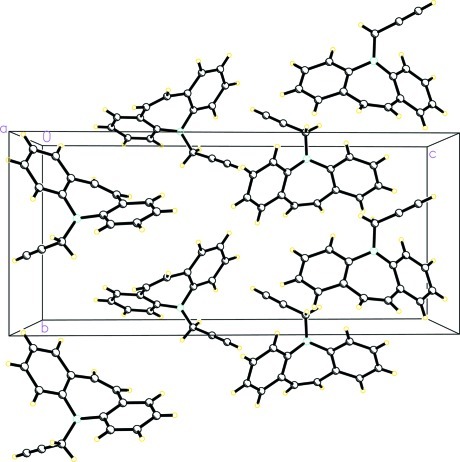
The crystal packing of the title compound viewed along the a axis.

## supplementary crystallographic information

### Comment

Iminostilbene (5*H*-dibenzo[b,f]azepine) was found to be a valuable intermediate in medicinal chemistry. It is also a basic nucleus of many anticonvulsant drugs including carbamazepine (Balaure *et al.*, 2009), oxazepine (Fuenfschilling *et al.*, 2005) and imipramine (Bhatt & Patel, 2005). The structural analogue of iminostilbene showed a wide range of biological activities such as antioxidant (Kumar & Naik, 2010), antihypertensive (Arya *et al.*, 1977), antitumor (Brzozowski & Saczewski, 2002), pesticidal (Kulkarni *et al.*, 1991), and DHFR inhibitory activity (Rosowsky *et al.*, 2004). The title compound was synthesized as part of our ongoing research on the synthesis of new derivatives of iminostilbene as potential anti-epileptic agents with more efficacy, low toxicity and no side effects as compared to the available drugs (Sadashiva *et al.*, 2005).

The structure of the title compound contains two independent molecules in the asymmetric unit (Fig. 1). Each molecule is consists of two phenyl rings fused to a seven-membered azepine ring adopting a boat conformation. The overall shape of each molecule is butterfly-like. The dihedral angles between the phenyl rings are 46.95 (11) and 52.21 (11)° for C1–C5/C14, C6–C11 and C18–C22/C31, C23–C28, respectively. The bond lengths and angles are found to be similar to those observed in other structurally related compounds (Jayasankar *et al.*, 2009; Nagaraj *et al.*, 2005; Sadashiva *et al.*, 2005). The crystal packing (Fig. 2) is enforced only by van der Waals forces.

### Experimental

To the stirred solution of iminostilbene (0.2921 g, 1.5 mmol) in DMF (3.0 mL), propargyl bromide (0.41 ml, 4.5 mmol) and potassium carbonate (0.418 ml, 3.02 mmol) were added and refluxed for 15 h at 90°C. After completion of the reaction reaction as judged by TLC, the crude mixture was adsorbed on silica and purified by column chromatography (eluent: n-hexane:ethyl acetate, 9.5:0.5 *v*/*v*) to obtain the title compound in 77% yield. Recrystallization by slow evaporation of a methanol solution afforded yellow crystals suitable for single-crystal X-ray diffraction studies. Iminostilbene and propargyl bromide were purchased from Alfa Acer and Aldrich, respectively.

### Refinement

Aromatic, methine and methylene H atoms were positioned geometrically with C—H = 0.93–0.97 Å, and constrained to ride on their parent atoms with *U*_iso_(H) = 1.2*U*_eq_(CH). The hydrogen of the acetylene groups (C–H= 0.91 (2) and 0.92 (3) Å) were located in difference Fourier maps and refined isotropically.

### Crystal data

**Table:**

C_17_H_13_N	*F*(000) = 976
*M**_r_* = 231.28	*D*_x_ = 1.202 Mg m^−^^3^
Monoclinic, *P*2_1_/*c*	Mo *K*α radiation, λ = 0.71073 Å
Hall symbol: -P 2ybc	Cell parameters from 2353 reflections
*a* = 11.4406 (5) Å	θ = 2.5–20.5°
*b* = 10.0256 (4) Å	µ = 0.07 mm^−^^1^
*c* = 22.3155 (10) Å	*T* = 273 K
β = 92.910 (1)°	Block, yellow
*V* = 2556.26 (19) Å^3^	0.36 × 0.19 × 0.15 mm
*Z* = 8	

### Data collection

**Table:**

Bruker SMART APEX CCD area-detector diffractometer	4760 independent reflections
Radiation source: fine-focus sealed tube	2894 reflections with *I* > 2σ(*I*)
Graphite monochromator	*R*_int_ = 0.031
ω scan	θ_max_ = 25.5°, θ_min_ = 1.8°
Absorption correction: multi-scan (*SADABS*; Bruker, 2000)	*h* = −13→13
*T*_min_ = 0.975, *T*_max_ = 0.990	*k* = −12→12
14791 measured reflections	*l* = −25→27

### Refinement

**Table:**

Refinement on *F*^2^	Primary atom site location: structure-invariant direct methods
Least-squares matrix: full	Secondary atom site location: difference Fourier map
*R*[*F*^2^ > 2σ(*F*^2^)] = 0.048	Hydrogen site location: inferred from neighbouring sites
*wR*(*F*^2^) = 0.129	H atoms treated by a mixture of independent and constrained refinement
*S* = 1.02	*w* = 1/[σ^2^(*F*_o_^2^) + (0.0567*P*)^2^ + 0.1752*P*] where *P* = (*F*_o_^2^ + 2*F*_c_^2^)/3
4760 reflections	(Δ/σ)_max_ < 0.001
333 parameters	Δρ_max_ = 0.13 e Å^−^^3^
0 restraints	Δρ_min_ = −0.17 e Å^−^^3^

### Special details

**Table:**

Geometry. All e.s.d.'s (except the e.s.d. in the dihedral angle between two l.s. planes) are estimated using the full covariance matrix. The cell e.s.d.'s are taken into account individually in the estimation of e.s.d.'s in distances, angles and torsion angles; correlations between e.s.d.'s in cell parameters are only used when they are defined by crystal symmetry. An approximate (isotropic) treatment of cell e.s.d.'s is used for estimating e.s.d.'s involving l.s. planes.
Refinement. Refinement of *F*^2^ against ALL reflections. The weighted *R*-factor *wR* and goodness of fit *S* are based on *F*^2^, conventional *R*-factors *R* are based on *F*, with *F* set to zero for negative *F*^2^. The threshold expression of *F*^2^ > σ(*F*^2^) is used only for calculating *R*-factors(gt) *etc*. and is not relevant to the choice of reflections for refinement. *R*-factors based on *F*^2^ are statistically about twice as large as those based on *F*, and *R*- factors based on ALL data will be even larger.

### Fractional atomic coordinates and isotropic or equivalent isotropic displacement parameters (Å^2^)

**Table:**

	*x*	*y*	*z*	*U*_iso_*/*U*_eq_	
N1	0.35570 (12)	0.40653 (14)	0.17436 (6)	0.0525 (4)	
N2	0.10076 (12)	0.57540 (14)	−0.14279 (7)	0.0564 (4)	
C1	0.3841 (3)	0.1387 (3)	0.06172 (12)	0.0989 (9)	
H1A	0.3522	0.0556	0.0520	0.119*	
C2	0.4688 (3)	0.1918 (4)	0.02656 (12)	0.1181 (13)	
H2A	0.4935	0.1447	−0.0064	0.142*	
C3	0.5156 (3)	0.3127 (4)	0.04036 (12)	0.1084 (10)	
H3A	0.5714	0.3490	0.0162	0.130*	
C4	0.4818 (2)	0.3821 (2)	0.08950 (10)	0.0771 (6)	
H4A	0.5161	0.4642	0.0988	0.092*	
C5	0.39677 (17)	0.33144 (19)	0.12571 (8)	0.0578 (5)	
C6	0.37342 (15)	0.35079 (17)	0.23312 (8)	0.0514 (5)	
C7	0.44563 (17)	0.4125 (2)	0.27675 (9)	0.0643 (5)	
H7A	0.4914	0.4848	0.2664	0.077*	
C8	0.4501 (2)	0.3673 (2)	0.33551 (10)	0.0757 (6)	
H8A	0.4980	0.4099	0.3645	0.091*	
C9	0.3841 (2)	0.2598 (2)	0.35101 (10)	0.0780 (6)	
H9A	0.3852	0.2306	0.3906	0.094*	
C10	0.31622 (19)	0.1957 (2)	0.30754 (10)	0.0707 (6)	
H10A	0.2731	0.1215	0.3181	0.085*	
C11	0.30968 (16)	0.23809 (18)	0.24806 (9)	0.0570 (5)	
C12	0.2357 (2)	0.1663 (2)	0.20414 (11)	0.0782 (7)	
H12A	0.1695	0.1254	0.2183	0.094*	
C13	0.2524 (2)	0.1527 (2)	0.14597 (12)	0.0834 (7)	
H13A	0.1974	0.1010	0.1244	0.100*	
C14	0.3450 (2)	0.2079 (2)	0.11208 (9)	0.0697 (6)	
C15	0.36741 (17)	0.55153 (18)	0.17127 (9)	0.0644 (5)	
H15A	0.3342	0.5912	0.2062	0.077*	
H15B	0.4498	0.5748	0.1720	0.077*	
C16	0.30875 (19)	0.6063 (2)	0.11707 (10)	0.0678 (6)	
C17	0.2623 (2)	0.6535 (3)	0.07470 (13)	0.0897 (8)	
H17A	0.224 (2)	0.690 (3)	0.0417 (11)	0.125 (10)*	
C18	0.2287 (2)	0.8931 (2)	−0.08634 (13)	0.0902 (8)	
H18A	0.2908	0.9494	−0.0940	0.108*	
C19	0.1591 (3)	0.9229 (3)	−0.04040 (14)	0.1062 (10)	
H19A	0.1734	0.9993	−0.0175	0.127*	
C20	0.0687 (3)	0.8409 (3)	−0.02805 (11)	0.0979 (9)	
H20A	0.0209	0.8613	0.0032	0.117*	
C21	0.0478 (2)	0.7265 (2)	−0.06211 (10)	0.0759 (6)	
H21A	−0.0135	0.6701	−0.0533	0.091*	
C22	0.11748 (17)	0.69593 (18)	−0.10896 (9)	0.0576 (5)	
C23	0.05986 (16)	0.59232 (17)	−0.20371 (8)	0.0535 (5)	
C24	−0.04889 (18)	0.5457 (2)	−0.22448 (10)	0.0688 (6)	
H24A	−0.0993	0.5092	−0.1975	0.083*	
C25	−0.0833 (2)	0.5525 (2)	−0.28392 (11)	0.0818 (7)	
H25A	−0.1562	0.5198	−0.2971	0.098*	
C26	−0.0107 (3)	0.6074 (2)	−0.32395 (11)	0.0859 (7)	
H26A	−0.0342	0.6122	−0.3644	0.103*	
C27	0.0972 (2)	0.65544 (19)	−0.30433 (10)	0.0769 (6)	
H27A	0.1462	0.6926	−0.3319	0.092*	
C28	0.13471 (17)	0.64939 (17)	−0.24365 (9)	0.0591 (5)	
C29	0.25089 (19)	0.6983 (2)	−0.22446 (11)	0.0739 (6)	
H29A	0.3091	0.6891	−0.2518	0.089*	
C30	0.28259 (18)	0.7546 (2)	−0.17196 (12)	0.0773 (7)	
H30A	0.3605	0.7805	−0.1667	0.093*	
C31	0.20917 (18)	0.78001 (19)	−0.12236 (10)	0.0645 (6)	
C32	0.05539 (18)	0.45998 (19)	−0.11137 (9)	0.0681 (6)	
H32A	0.0450	0.3860	−0.1391	0.082*	
H32B	−0.0203	0.4817	−0.0963	0.082*	
C33	0.1359 (2)	0.4210 (2)	−0.06140 (10)	0.0707 (6)	
C34	0.2009 (3)	0.3889 (3)	−0.02264 (13)	0.0962 (8)	
H34A	0.252 (3)	0.361 (3)	0.0083 (13)	0.148 (12)*	

### Atomic displacement parameters (Å^2^)

**Table:**

	*U*^11^	*U*^22^	*U*^33^	*U*^12^	*U*^13^	*U*^23^
N1	0.0636 (9)	0.0430 (8)	0.0517 (9)	−0.0030 (7)	0.0095 (7)	−0.0013 (7)
N2	0.0611 (9)	0.0493 (9)	0.0591 (10)	−0.0075 (7)	0.0062 (8)	0.0061 (8)
C1	0.149 (3)	0.0786 (17)	0.0660 (17)	0.0438 (17)	−0.0289 (17)	−0.0157 (14)
C2	0.164 (3)	0.136 (3)	0.0529 (17)	0.089 (3)	−0.0006 (19)	−0.0074 (19)
C3	0.109 (2)	0.149 (3)	0.0688 (18)	0.068 (2)	0.0261 (15)	0.0170 (19)
C4	0.0741 (14)	0.0982 (16)	0.0600 (14)	0.0252 (13)	0.0154 (12)	0.0100 (13)
C5	0.0647 (12)	0.0612 (12)	0.0474 (11)	0.0154 (10)	0.0010 (10)	0.0009 (10)
C6	0.0530 (10)	0.0502 (10)	0.0516 (11)	0.0057 (9)	0.0095 (9)	−0.0038 (9)
C7	0.0680 (12)	0.0599 (12)	0.0651 (13)	0.0002 (10)	0.0047 (11)	−0.0076 (10)
C8	0.0884 (16)	0.0767 (15)	0.0608 (14)	0.0191 (13)	−0.0061 (12)	−0.0133 (12)
C9	0.0987 (17)	0.0787 (15)	0.0577 (14)	0.0303 (14)	0.0149 (13)	0.0067 (12)
C10	0.0766 (14)	0.0637 (13)	0.0734 (15)	0.0110 (11)	0.0211 (12)	0.0151 (11)
C11	0.0597 (11)	0.0499 (10)	0.0621 (12)	0.0041 (9)	0.0102 (10)	0.0060 (9)
C12	0.0802 (15)	0.0585 (12)	0.0955 (18)	−0.0184 (11)	−0.0002 (14)	0.0123 (12)
C13	0.1049 (18)	0.0552 (13)	0.0867 (18)	−0.0142 (12)	−0.0278 (16)	−0.0035 (12)
C14	0.0937 (16)	0.0578 (12)	0.0560 (13)	0.0169 (12)	−0.0129 (12)	−0.0048 (11)
C15	0.0732 (13)	0.0506 (11)	0.0703 (14)	−0.0054 (10)	0.0119 (11)	0.0018 (10)
C16	0.0720 (13)	0.0571 (12)	0.0759 (15)	0.0036 (11)	0.0207 (12)	0.0087 (11)
C17	0.0928 (18)	0.0917 (18)	0.0863 (19)	0.0256 (14)	0.0204 (15)	0.0225 (15)
C18	0.0917 (18)	0.0600 (14)	0.114 (2)	−0.0077 (13)	−0.0405 (17)	0.0088 (15)
C19	0.134 (3)	0.0695 (17)	0.109 (2)	0.0161 (18)	−0.051 (2)	−0.0141 (16)
C20	0.124 (2)	0.0891 (18)	0.0779 (17)	0.0395 (18)	−0.0167 (16)	−0.0164 (15)
C21	0.0812 (15)	0.0737 (15)	0.0724 (15)	0.0120 (12)	0.0008 (13)	−0.0028 (12)
C22	0.0572 (12)	0.0541 (11)	0.0604 (13)	0.0046 (10)	−0.0070 (10)	0.0053 (10)
C23	0.0578 (11)	0.0430 (10)	0.0599 (12)	0.0061 (9)	0.0051 (10)	−0.0001 (9)
C24	0.0649 (13)	0.0697 (13)	0.0716 (14)	−0.0026 (10)	0.0015 (11)	−0.0085 (11)
C25	0.0873 (16)	0.0726 (15)	0.0835 (17)	0.0015 (13)	−0.0152 (14)	−0.0139 (13)
C26	0.124 (2)	0.0647 (14)	0.0671 (15)	0.0149 (15)	−0.0145 (16)	−0.0056 (12)
C27	0.1099 (19)	0.0537 (12)	0.0688 (15)	0.0136 (13)	0.0202 (14)	0.0115 (11)
C28	0.0664 (13)	0.0448 (10)	0.0671 (13)	0.0103 (9)	0.0126 (11)	0.0090 (9)
C29	0.0647 (14)	0.0627 (13)	0.0965 (18)	0.0107 (11)	0.0263 (13)	0.0264 (13)
C30	0.0504 (12)	0.0679 (14)	0.113 (2)	−0.0065 (11)	−0.0060 (14)	0.0334 (14)
C31	0.0581 (12)	0.0525 (12)	0.0811 (15)	−0.0037 (10)	−0.0157 (12)	0.0121 (11)
C32	0.0734 (13)	0.0601 (12)	0.0718 (14)	−0.0131 (11)	0.0123 (11)	0.0080 (11)
C33	0.0866 (15)	0.0601 (13)	0.0671 (14)	0.0002 (11)	0.0198 (13)	0.0117 (11)
C34	0.111 (2)	0.0952 (19)	0.0827 (19)	0.0185 (16)	0.0100 (17)	0.0232 (15)

### Geometric parameters (Å, º)

**Table:**

N1—C5	1.420 (2)	C15—H15B	0.9700
N1—C6	1.431 (2)	C16—C17	1.162 (3)
N1—C15	1.462 (2)	C17—H17A	0.91 (2)
N2—C23	1.425 (2)	C18—C19	1.362 (4)
N2—C22	1.432 (2)	C18—C31	1.401 (3)
N2—C32	1.462 (2)	C18—H18A	0.9300
C1—C2	1.385 (4)	C19—C20	1.361 (4)
C1—C14	1.413 (3)	C19—H19A	0.9300
C1—H1A	0.9300	C20—C21	1.390 (3)
C2—C3	1.354 (4)	C20—H20A	0.9300
C2—H2A	0.9300	C21—C22	1.381 (3)
C3—C4	1.371 (3)	C21—H21A	0.9300
C3—H3A	0.9300	C22—C31	1.390 (3)
C4—C5	1.392 (3)	C23—C24	1.387 (3)
C4—H4A	0.9300	C23—C28	1.390 (3)
C5—C14	1.399 (3)	C24—C25	1.366 (3)
C6—C7	1.390 (2)	C24—H24A	0.9300
C6—C11	1.394 (2)	C25—C26	1.366 (3)
C7—C8	1.386 (3)	C25—H25A	0.9300
C7—H7A	0.9300	C26—C27	1.376 (3)
C8—C9	1.371 (3)	C26—H26A	0.9300
C8—H8A	0.9300	C27—C28	1.401 (3)
C9—C10	1.371 (3)	C27—H27A	0.9300
C9—H9A	0.9300	C28—C29	1.460 (3)
C10—C11	1.392 (3)	C29—C30	1.334 (3)
C10—H10A	0.9300	C29—H29A	0.9300
C11—C12	1.453 (3)	C30—C31	1.446 (3)
C12—C13	1.329 (3)	C30—H30A	0.9300
C12—H12A	0.9300	C32—C33	1.463 (3)
C13—C14	1.444 (3)	C32—H32A	0.9700
C13—H13A	0.9300	C32—H32B	0.9700
C15—C16	1.460 (3)	C33—C34	1.157 (3)
C15—H15A	0.9700	C34—H34A	0.92 (3)
			
C5—N1—C6	117.16 (14)	H15A—C15—H15B	107.9
C5—N1—C15	117.16 (15)	C17—C16—C15	178.0 (2)
C6—N1—C15	114.99 (14)	C16—C17—H17A	178.5 (18)
C23—N2—C22	115.52 (13)	C19—C18—C31	121.8 (3)
C23—N2—C32	116.38 (14)	C19—C18—H18A	119.1
C22—N2—C32	117.14 (15)	C31—C18—H18A	119.1
C2—C1—C14	121.4 (3)	C20—C19—C18	119.9 (3)
C2—C1—H1A	119.3	C20—C19—H19A	120.0
C14—C1—H1A	119.3	C18—C19—H19A	120.0
C3—C2—C1	119.7 (3)	C19—C20—C21	120.0 (3)
C3—C2—H2A	120.1	C19—C20—H20A	120.0
C1—C2—H2A	120.1	C21—C20—H20A	120.0
C2—C3—C4	120.8 (3)	C22—C21—C20	120.5 (2)
C2—C3—H3A	119.6	C22—C21—H21A	119.8
C4—C3—H3A	119.6	C20—C21—H21A	119.8
C3—C4—C5	120.9 (3)	C21—C22—C31	119.9 (2)
C3—C4—H4A	119.6	C21—C22—N2	121.30 (18)
C5—C4—H4A	119.6	C31—C22—N2	118.7 (2)
C4—C5—C14	119.8 (2)	C24—C23—C28	119.72 (18)
C4—C5—N1	121.21 (18)	C24—C23—N2	121.62 (18)
C14—C5—N1	118.85 (19)	C28—C23—N2	118.52 (16)
C7—C6—C11	119.52 (17)	C25—C24—C23	121.2 (2)
C7—C6—N1	121.33 (16)	C25—C24—H24A	119.4
C11—C6—N1	118.96 (15)	C23—C24—H24A	119.4
C8—C7—C6	120.6 (2)	C24—C25—C26	120.0 (2)
C8—C7—H7A	119.7	C24—C25—H25A	120.0
C6—C7—H7A	119.7	C26—C25—H25A	120.0
C9—C8—C7	120.1 (2)	C25—C26—C27	119.9 (2)
C9—C8—H8A	120.0	C25—C26—H26A	120.0
C7—C8—H8A	120.0	C27—C26—H26A	120.0
C10—C9—C8	119.3 (2)	C26—C27—C28	121.2 (2)
C10—C9—H9A	120.4	C26—C27—H27A	119.4
C8—C9—H9A	120.4	C28—C27—H27A	119.4
C9—C10—C11	122.2 (2)	C23—C28—C27	117.99 (19)
C9—C10—H10A	118.9	C23—C28—C29	122.09 (18)
C11—C10—H10A	118.9	C27—C28—C29	119.9 (2)
C10—C11—C6	118.14 (18)	C30—C29—C28	127.0 (2)
C10—C11—C12	119.56 (19)	C30—C29—H29A	116.5
C6—C11—C12	122.28 (18)	C28—C29—H29A	116.5
C13—C12—C11	127.0 (2)	C29—C30—C31	127.2 (2)
C13—C12—H12A	116.5	C29—C30—H30A	116.4
C11—C12—H12A	116.5	C31—C30—H30A	116.4
C12—C13—C14	128.2 (2)	C22—C31—C18	117.9 (2)
C12—C13—H13A	115.9	C22—C31—C30	122.15 (19)
C14—C13—H13A	115.9	C18—C31—C30	120.0 (2)
C5—C14—C1	117.4 (2)	N2—C32—C33	110.60 (16)
C5—C14—C13	122.65 (19)	N2—C32—H32A	109.5
C1—C14—C13	119.9 (2)	C33—C32—H32A	109.5
C16—C15—N1	111.94 (16)	N2—C32—H32B	109.5
C16—C15—H15A	109.2	C33—C32—H32B	109.5
N1—C15—H15A	109.2	H32A—C32—H32B	108.1
C16—C15—H15B	109.2	C34—C33—C32	178.7 (3)
N1—C15—H15B	109.2	C33—C34—H34A	178 (2)
			
C14—C1—C2—C3	−0.1 (4)	C31—C18—C19—C20	0.8 (4)
C1—C2—C3—C4	1.3 (4)	C18—C19—C20—C21	0.3 (4)
C2—C3—C4—C5	−1.3 (4)	C19—C20—C21—C22	−0.7 (3)
C3—C4—C5—C14	0.1 (3)	C20—C21—C22—C31	−0.1 (3)
C3—C4—C5—N1	−175.84 (18)	C20—C21—C22—N2	177.06 (17)
C6—N1—C5—C4	−118.89 (18)	C23—N2—C22—C21	112.72 (19)
C15—N1—C5—C4	24.0 (2)	C32—N2—C22—C21	−30.2 (2)
C6—N1—C5—C14	65.1 (2)	C23—N2—C22—C31	−70.1 (2)
C15—N1—C5—C14	−152.05 (16)	C32—N2—C22—C31	146.96 (17)
C5—N1—C6—C7	115.39 (19)	C22—N2—C23—C24	−115.27 (19)
C15—N1—C6—C7	−28.3 (2)	C32—N2—C23—C24	28.0 (2)
C5—N1—C6—C11	−69.7 (2)	C22—N2—C23—C28	68.9 (2)
C15—N1—C6—C11	146.70 (17)	C32—N2—C23—C28	−147.85 (17)
C11—C6—C7—C8	−3.4 (3)	C28—C23—C24—C25	1.2 (3)
N1—C6—C7—C8	171.53 (17)	N2—C23—C24—C25	−174.52 (17)
C6—C7—C8—C9	0.8 (3)	C23—C24—C25—C26	−0.8 (3)
C7—C8—C9—C10	1.7 (3)	C24—C25—C26—C27	0.1 (3)
C8—C9—C10—C11	−1.6 (3)	C25—C26—C27—C28	0.0 (3)
C9—C10—C11—C6	−1.0 (3)	C24—C23—C28—C27	−1.1 (3)
C9—C10—C11—C12	−179.7 (2)	N2—C23—C28—C27	174.82 (16)
C7—C6—C11—C10	3.4 (3)	C24—C23—C28—C29	−179.05 (18)
N1—C6—C11—C10	−171.60 (16)	N2—C23—C28—C29	−3.2 (3)
C7—C6—C11—C12	−177.87 (18)	C26—C27—C28—C23	0.5 (3)
N1—C6—C11—C12	7.1 (3)	C26—C27—C28—C29	178.50 (19)
C10—C11—C12—C13	−149.7 (2)	C23—C28—C29—C30	−34.1 (3)
C6—C11—C12—C13	31.6 (3)	C27—C28—C29—C30	147.9 (2)
C11—C12—C13—C14	−1.8 (4)	C28—C29—C30—C31	0.0 (3)
C4—C5—C14—C1	1.0 (3)	C21—C22—C31—C18	1.2 (3)
N1—C5—C14—C1	177.08 (16)	N2—C22—C31—C18	−176.05 (16)
C4—C5—C14—C13	−176.86 (19)	C21—C22—C31—C30	−178.21 (17)
N1—C5—C14—C13	−0.8 (3)	N2—C22—C31—C30	4.6 (3)
C2—C1—C14—C5	−1.1 (3)	C19—C18—C31—C22	−1.6 (3)
C2—C1—C14—C13	176.9 (2)	C19—C18—C31—C30	177.8 (2)
C12—C13—C14—C5	−32.8 (3)	C29—C30—C31—C22	33.3 (3)
C12—C13—C14—C1	149.4 (2)	C29—C30—C31—C18	−146.1 (2)
C5—N1—C15—C16	57.1 (2)	C23—N2—C32—C33	155.99 (17)
C6—N1—C15—C16	−159.25 (16)	C22—N2—C32—C33	−61.4 (2)
